# Targeted therapy in lymphoma

**DOI:** 10.1186/1756-8722-3-45

**Published:** 2010-11-23

**Authors:** Patrick B Johnston, RuiRong Yuan, Franco Cavalli, Thomas E Witzig

**Affiliations:** 1 Mayo Clinic, Rochester, MN, USA; 2 Novartis Pharmaceuticals, Florham Park, NJ, and New Jersey Medical School (UMDNJ), Newark, NJ, USA; 3 Oncology Institute of Southern Switzerland (IOSI), Bellinzona, Switzerland

## Abstract

Discovery of new treatments for lymphoma that prolong survival and are less toxic than currently available agents represents an urgent unmet need. We now have a better understanding of the molecular pathogenesis of lymphoma, such as aberrant signal transduction pathways, which have led to the discovery and development of targeted therapeutics. The ubiquitin-proteasome and the Akt/mammalian target of rapamycin (mTOR) pathways are examples of pathological mechanisms that are being targeted in drug development efforts. Bortezomib (a small molecule protease inhibitor) and the mTOR inhibitors temsirolimus, everolimus, and ridaforolimus are some of the targeted therapies currently being studied in the treatment of aggressive, relapsed/refractory lymphoma. This review will discuss the rationale for and summarize the reported findings of initial and ongoing investigations of mTOR inhibitors and other small molecule targeted therapies in the treatment of lymphoma.

## Introduction

Despite remarkable advances in diagnosis and treatment, lymphoma continues to rank as a leading cause of cancer-related mortality. Recent cancer statistics for the United States project non-Hodgkin lymphoma (NHL) to be the sixth most commonly diagnosed cancer in 2010 in both men and women, and the eighth and sixth leading cause of cancer-related death in men and women, respectively [1]. Based on data from national cancer registries, 65,540 new cases of NHL and 20,210 deaths from NHL are estimated to occur in 2010. In contrast, Hodgkin lymphoma (HL) is less common (8,490 estimated new cases in 2010) and is associated with fewer deaths (1,320 estimated deaths in 2010) [1]. In the European Union, reported NHL estimates for the year 2006 were even higher, with 72,800 new cases and 33,000 deaths [2].

Current treatments for NHL are not optimally effective, with relapse and resistance to chemotherapy common and the risk of secondary malignancies an ongoing concern. Long-term prognosis in patients who relapse with aggressive NHL, such as diffuse large B-cell lymphoma (DLBCL) and mantle cell lymphoma (MCL), after induction therapy typically is dismal [3,4]. Discovery of new treatments that prolong survival and are less toxic represents an urgent unmet medical need. Intensive research efforts that were focused on better understanding the molecular pathogenesis of lymphoma have paved the way toward identifying and testing targeted therapeutics [5].

Delineation of signal transduction mechanisms involved in the pathogenesis of lymphoma has revealed new therapeutic targets for clinical investigation (Table 1) [6-14]. For example, the ubiquitin-proteasome signaling pathway, which is a fundamental component of cellular proliferation and survival, mediates the degradation of proteins involved in the regulation of cell growth [15]. The proteasome activates nuclear factor-κB (NF-κB) signaling by degrading IκB kinase (eg, the NF-κB inhibitory protein), resulting in the promotion of tumor growth and metastasis [15]. Elucidation of this regulatory signaling pathway identified IκB kinase as a molecular target for development of drugs with activity against lymphoma. Bortezomib (Velcade^®^) is the prototype small-molecule protease inhibitor that is approved for the treatment of relapsed/refractory MCL and multiple myeloma [15,16].

**Table 1:** Investigational therapeutic targets in lymphoma treatment

Pathway/Protein	Oncogenic Mechanism	Molecular Target(s)	Drug Class	Investigational Drugs in Clinical Trials
Ubiquitin-proteasome pathway [6,7]	Dysregulation of intracellular cell cycle proteins	NF-κB inhibitory protein (IκB)	Small-molecule proteasome inhibitors	Bortezomib (PS-341, Velcade™)

Akt/mTOR pathway [8-10]	Aberrant activation of mTOR-mediated regulation of cell growth, proliferation, apoptosis, angiogenesis, nutrient uptake	mTORC1 (mTORC2?)	mTOR inhibitors	Temsirolimus (CCI-779, Torisel^®^) Everolimus (RAD001, Afinitor^®^) Ridaforolimus (formerly deforolimus, AP23573)

Cell-mediated immunity, cytokines [11]	Aberrant activation of prosurvival cytokines and cellular immune response	TNF-α, IL-6, IL-8, and VEGF; T cells and NK cells	Immunomodulatory drugs (IMiDs)	Lenalidomide (Revlimid^®^)

VEGF receptors, PDGF receptors [12,13]	Tumor proliferation, angiogenesis	Tyrosine kinase	Tyrosine kinase inhibitors	Sunitinib (SU11248, Sutent^®^) Sorafenib (Nexavar^®^)

Histone deacetylase [14]	Dysregulated histone deacetylation in promoters of growth regulatory genes (gene silencing)	Histone deacetylase	Histone deacetylase inhibitors (HDACIs)	Vorinostat (Zolinza^®^) Romidepsin (FK228) Valproic acid Panobinostat (LBH589) Others

Abbreviations: IL-6 = interleukin-6; IL-8 = interleukin-8; mTOR = mammalian target of rapamycin; PDGF = platelet-derived growth factor; PI3K = phosphoinositide 3-kinase; TNF-α = tumor necrosis factor-alpha; VEGF = vascular endothelial growth factor.

**Figure 1:**
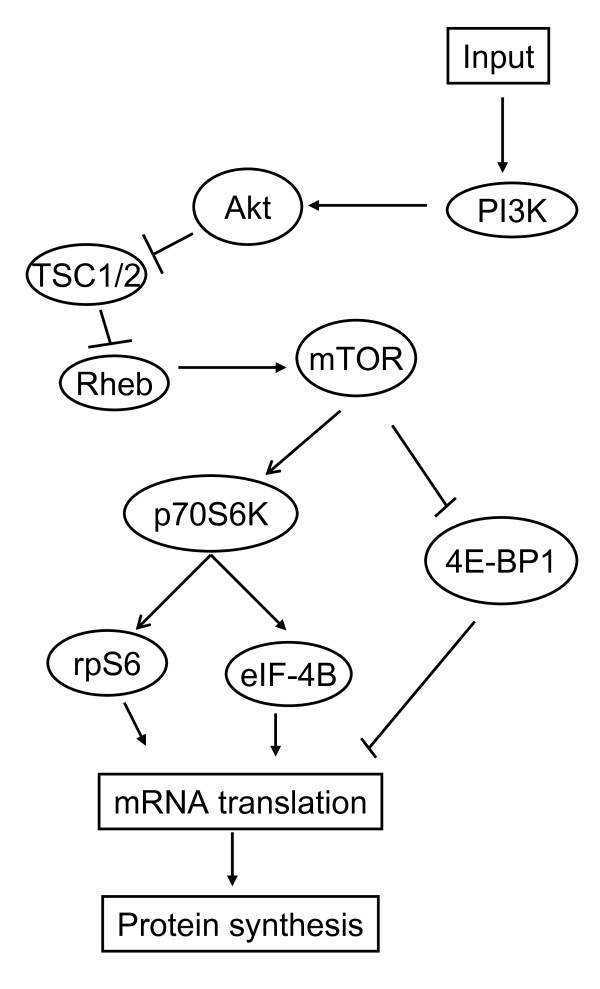
**The PI3K/Akt signaling pathway**. Reprinted with permission from Altman JK, Platanias LC: Exploiting the mammalian target of rapamycin pathway in hematologic malignancies. *Curr Opin Hematol*. 2008, **15**:88-94.

The phosphoinositide 3-kinase (PI3K)/Akt signaling pathway (Figure 1) is another important signal transduction pathway that is aberrantly activated in various different types of cancer, including many hematologic malignancies [8]. PI3K is a lipid kinase that is activated by a variety of cellular input signals, such as growth factor receptor tyrosine kinase stimulation. Activated PI3K enables recruitment of the serine/threonine kinase Akt to the cell membrane where it undergoes phosphorylation. Phosphorylated Akt subsequently activates several other intracellular signaling proteins [8]. One downstream target of Akt is the mammalian target of rapamycin (mTOR), a cytoplasmic serine/threonine kinase that, when activated, promotes mRNA translation and protein synthesis, resulting in the regulation of cell growth and proliferation, cellular metabolism, and angiogenesis [8]. The mTOR pathway is aberrantly activated in many hematologic malignancies, including some forms of NHL and HL [8-10]. The mTOR inhibitors everolimus (Afinitor^®^) and temsirolimus (Torisel^®^) are currently under clinical investigation for the treatment of NHL and HL, and ridaforolimus (formerly deforolimus) is being evaluated in patients with hematological malignancies including lymphoma.

Other investigational targeted therapies are of interest in the treatment of NHL and HL (Table 1). Lenalidomide (Revlimid^®^) is a derivative of thalidomide that is approved for use in combination with dexamethasone for the treatment of previously treated multiple myeloma [17]. Lenalidomide is currently being investigated in a variety of solid tumors and other hematologic malignancies, including lymphoma [17]. While the exact mechanism is not known, lenalidomide is believed to exert anti-metastatic, anti-proliferative, and immunomodulatory activities [11,17]. Sunitinib (Sutent^®^) and sorafenib (Nexavar^®^) are tyrosine kinase inhibitors that interrupt tumor proliferation and angiogenesis by inhibiting vascular endothelial growth factor (VEGF) and platelet-derived growth factor (PDGF) receptors [12,13]. The histone deacetylase inhibitors (Table 1) represent an emerging therapeutic approach that targets aberrant gene expression, putatively blocking the development of malignant phenotypes (eg, epigenetic therapy) [14,18]. Histones are structural proteins involved in the expression of genes that regulate tumor cell differentiation and apoptosis [14,18]. Vorinostat (Zolinza^®^), romidepsin (FK228), valproic acid, and panobinostat (LBH589) are some of the histone deacetylase inhibitors (HDACIs) currently being investigated for clinical activity [14,18,19].

Herein we review the experience with targeted treatments for lymphoma that have advanced from phase I to phase III clinical trials. We will focus our discussion primarily on published data in NHL, including MCL and DLBCL. It is hoped that the wealth of information being discovered in the molecular pathogenesis of lymphoma and the development of targeted therapeutics for these aberrant pathways will identify highly specific, less toxic agents for the treatment of lymphomas.

### Small-molecule proteasome inhibitors

The clinical trial experience to date for bortezomib treatment of lymphoma includes studies of mixed lymphoma populations and studies that limited enrollment to patients with MCL, DLBCL, or HL (Table 2) [20-33].

**Table 2:** Clinical trial experience with bortezomib in lymphoma

Reference	Study	Evaluable Patients	ORR (CR + PR)
**Treatment-naïve MCL**			

Kahl et al 2008 [20]	Phase II, single-arm, VcR-CVAD followed by maintenance rituximab therapy	N = 30	90%

**Relapsed/refractory MCL (and other lymphomas)**			

O'Connor et al 2005 [21]	Phase II, single-arm, monotherapy (1.5 mg/m^2 ^days 1, 4, 8, 11 every 21 days)	N = 24: MCL (n = 10), follicular lymphoma (n = 9), small lymphocytic lymphoma or CLL (n = 3), marginal zone lymphoma (n = 2)	MCL 50% Follicular lymphoma 78% Small lymphocytic lymphoma or CLL 0% Marginal zone lymphoma 100%

Gerecitano et al 2009 [22]	Extension of O'Connor et al 2005 trial: continuing patients switched to weekly bortezomib 1.8 mg/m^2^	N = 22: MCL (n = 8), follicular lymphoma (n = 14)	MCL 25% Follicular lymphoma 14%

Goy et al 2005 [23]	Phase II, single-arm, monotherapy (1.5 mg/m^2 ^days 1, 4, 8, 11 every 21 days)	N = 50: MCL (n = 29), other B-cell lymphomas (n = 21)	MCL 41% Other B-cell lymphomas 19%

Strauss et al 2006 [24]	Phase II, single-arm, monotherapy (1.3 mg/m^2 ^days 1, 4, 8, 11 every 21 days)	N = 48: MCL (n = 24), follicular lymphoma (n = 11), other lymphomas (n = 13)	MCL 29% Follicular lymphoma 18% Others 23%

**Relapsed/refractory MCL**			

PINNACLE, Fisher et al 2006 [25]	Phase II, single-arm, monotherapy (1.3 mg/m^2 ^days 1, 4, 8, 11 every 21 days)	N = 141	33%

Updated PINNACLE,^a ^Goy et al 2009 [26]	Phase II, single-arm, monotherapy (1.3 mg/m^2 ^days 1, 4, 8, 11 every 21 days)	N = 55	29%

Belch et al 2007 [27]	Phase II, single-arm, monotherapy (1.3 mg/m^2 ^days 1, 4, 8, 11 every 21 days)	N = 28	46%

O'Connor et al 2009 [28]	Phase II, single-arm monotherapy (1.5 mg/m^2 ^days 1, 4, 8, 11 every 21 days)	N = 36^b^	47%

Weigert et al 2009 [29]	Multicenter observational study of R-HAD+B salvage regimen: - bortezomib (1.5 mg/m^2 ^days 1, 4) - cytarabine (2,000 mg/m^2 ^days 2, 3^c^) - dexamethasone (40 mg days 1-4) - Rituximab (375 mg/m^2 ^on day 0 for patients not refractory to prior rituximab-containing regimens)	N = 8	50%

**DLBCL**			

Dunleavy et al 2009 [30]	Phase I/II, 2-part study of bortezomib monotherapy (part A) followed by bortezomib plus DA-EPOCH (part B)	N = 47 (n = 23 part A, n = 44 part B)	Part A 4% Part B 34%

**Relapsed/refractory Hodgkin lymphoma**			

Trelle et al 2007 [31]	Phase II, bortezomib (1.3 mg/m^2^) plus dexamethasone (20 mg) on days 1, 4, 8, 11 every 21 days	N = 12	0% (17% SD, 83% PD)

Blum et al 2007 [32]	Phase II, single-arm, monotherapy (1.3 mg/m^2 ^on days 1, 4, 8, 11 every 21 days)	N = 29	0% (30% SD, 70% PD)

Mendler et al 2008 [33]	Phase II, single-arm bortezomib (1 mg/m^2 ^on days 1, 4, 8, 11) and gemcitabine (800 mg/m^2 ^on days 1, 8) every 21 days	N = 18	22%

Abbreviations: MCL - mantle-cell lymphoma, ORR - overall response rate, CR - complete response, PR - partial response, CLL - chronic lymphocytic leukemia, DLBCL - diffuse large B-cell lymphoma, DA-EPOCH - doxorubicin-based chemotherapy (etoposide, vincristine, doxorubicin, with cyclophosphamide and prednisone), R-HAD+B - bortezomib, high-dose cytarabine, dexamethasone, SD - stable disease, PD - progressive disease; VcR-CVAD - bortezomib, rituximab, cyclophosphamide, doxorubicin, vincristine and dexamethasone.

^a^Original PINNACLE publication reported data from median follow-up period of 13.4 months [25]; updated publication described data from median follow-up of 26.4 months [26]

^b^The 36 evaluable patients included 11 patients whose outcome was reported by O'Connor et al 2005 [21,28]

^c^Patients ≥60 years of age were treated with 1,000 mg/m^2 ^[29]

### Relapsed/refractory mantle cell lymphoma

Three phase II studies evaluated the safety and anti-tumor response of bortezomib in a total of 125 evaluable patients with various relapsed/refractory lymphomas (Table 2). Patients were heavily pretreated and had relapsed disease or tumors that were refractory to their most recent therapies. Roughly half (n = 63) of the evaluable patients in these 3 studies had MCL. Bortezomib was administered as monotherapy using a 21-day dosing cycle of 1.5 mg/m^2 ^or 1.3 mg/m^2 ^twice weekly for 2 weeks followed by a 1-week rest [21,23,24]. Overall response rates for the 1.5 mg/m^2 ^dose were 50% (1 unconfirmed complete response [uCR]/4 partial responses [PR]) [21] and 41% (6 CR/6 PR) [23]. Of the 24 evaluable patients who were treated with bortezomib 1.3 mg/m^2^, 29% achieved a measurable clinical response (1 CR/6 PR) [24]. Of 33 patients with MCL in one study, the median time to disease progression was 3.5 months, with an estimated progression-free survival at 6 months of 42% [23].

Three other studies examined the efficacy and safety of bortezomib in cohorts that consisted only of patients with MCL (Table 2). In the PINNACLE trial, bortezomib 1.3 mg/m^2 ^was administered to 141 evaluable patients according to the same 21-day cycle as in earlier studies, and 33% of patients responded to treatment (2 uCR/9 CR/36 PR) [25]. Although the median overall survival was not reached by the data cut-off point, 66% of patients remained alive after a median follow-up period of 13.4 months, and the 1-year survival probability was 94.3% for responding patients and 69.3% for all patients [25]. When the median follow-up was extended to 26.4 months, the median progression-free survival and median time to next treatment were, respectively, 20.3 and 23.9 months (complete responders), 9.7 and 13.3 months (partial responders), and 12.4 and 14.3 months (all responders) [26]. Findings from 2 smaller studies of bortezomib monotherapy in patients with MCL demonstrated overall response rates of 46% [27] and 47% [28].

Based on *in vitro *data showing synergy between bortezomib and conventional chemotherapy [34], Weigert and associates administered R-HAD+B, which is a novel regimen of bortezomib (1.5 mg/m^2 ^twice weekly every 21 days), high-dose cytarabine, and dexamethasone to 8 patients with advanced MCL (Table 2) [29]. Patients not refractory to prior rituximab regimens also received rituximab on day 0 [29]. Four patients were withdrawn from the study due to lack of response, but the 4 other patients completed 4 treatment cycles and achieved a CR (n = 2) or PR (n = 2) [29].

In addition to the studies combining bortezomib in the relapsed/refractory setting for NHL, 2 recent studies have assessed bortezomib in combination with other agents in previously untreated patients with NHL [20,35]. Bortezomib has been combined with rituximab, cyclophosphamide, doxorubicin, vincristine, and dexamethasone (VcR-CVAD) in the treatment of patients with untreated MCL in a phase II trial [20]. All patients achieving at least a PR after completing 6 cycles of the VcR-CVAD were offered maintenance rituximab therapy for 5 years. All 30 enrolled patients had completed the induction phase of the VcR-CVAD chemotherapy at the time of reporting. A 90% overall response rate was reported after VcR-CVAD with 77% CR/uCR and 13% PR with 10% of patients experiencing progressive disease during the induction chemotherapy. With a median follow-up of almost 18 months, the 18-month progression-free and overall survival was reported at 73% and 97%, respectively. Another trial incorporated bortezomib in combination with R-CHOP chemotherapy (rituximab, cyclophosphamide, hydroxydaunorubicin, vincristine, prednisone) in a phase I trial in patients with previously untreated aggressive NHL [35]. In this study, standard R-CHOP was given on a 21-day cycle and bortezomib was administered on days 1 and 4 of each cycle at 0.7 mg/m^2 ^(4 patients), 1.0 mg/m^2 ^(9 patients), or 1.3 mg/m^2 ^(7 patients). The histologic subtypes included both MCL and diffuse large B-cell lymphoma (DLBCL). The maximum tolerated dose was not reached and the 1.3 mg/m^2 ^dose was well tolerated. Neuropathy was a common side effect reported in 65% of patients [35].

Combination therapy with bortezomib is being evaluated further in an ongoing open-label, international phase III study. In this study, standard R-CHOP is being compared with a regimen of rituximab, cyclophosphamide, doxorubicin, bortezomib, and prednisone (VcR-CAP) in patients with newly diagnosed MCL who are not eligible for bone marrow transplantation (NCT00722137).

### Other lymphomas

Bortezomib monotherapy does not appear to have clinically meaningful anti-tumor activity in DLBCL, but when combined with chemotherapy, 34% of patients in one study responded to treatment (Table 2) [30]. Bortezomib also has been evaluated in patients with relapsed/refractory HL (Table 2), but none achieved a clinical response with bortezomib monotherapy [32] or with bortezomib plus dexamethasone [31]. A minimal clinical response (1 CR/3 PR) was observed with the combination of bortezomib and gemcitabine in 18 patients with DLBCL, but the investigators concluded that this combination should not be pursued due to grade 3/4 hepatotoxicity [33].

### Toxicity

Neutropenia and thrombocytopenia are common hematologic toxicities reported during twice-weekly bortezomib treatment [21,23,24,26,27,30]. Fatigue, peripheral neuropathy, and gastrointestinal disturbances were the most frequently reported non-hematologic adverse events associated with bortezomib [23,25-27]. The most common dose-limiting toxicities during treatment of MCL with twice-weekly bortezomib monotherapy (1.3 mg/m^2 ^or 1.5 mg/m^2^) were peripheral neuropathy, fatigue, and thrombocytopenia [21,23-25]. All of the 8 patients with advanced MCL who were treated with bortezomib plus high-dose cytarabine and dexamethasone developed grade 3/4 hematologic toxicity, 2 developed grade 3 febrile neutropenia, and 7 required G-CSF rescue [29]. In a continuation of one phase II monotherapy trial [21], Gerecitano and colleagues administered bortezomib monotherapy once-weekly (1.8 mg/m^2^) and concluded that weekly dosing is less toxic than the twice-weekly schedule but resulted in a lower clinical response rate (2 PR of 8 assessable patients with MCL) (Table 2) [22].

### mTOR Inhibitors

The rapamycin analogs everolimus and temsirolimus are mTOR inhibitors that have been approved for treatment of resistant renal cell carcinoma. Everolimus is administered orally, and temsirolimus intravenously. Based on *in vitro *activity of mTOR inhibitors in numerous lymphoma cell lines [36,37], both everolimus and temsirolimus have completed phase II clinical trials in NHL. Ridaforolimus and sirolimus are other mTOR inhibitors that also are in clinical testing for the treatment of lymphomas (Table 3) [38-46].

**Table 3:** Clinical trial experience with mTOR inhibitors in lymphoma

Reference	Study	Evaluable Patients	ORR (CR + PR)
**Relapsed/refractory MCL (and other lymphomas)**			

Everolimus [38]	Phase II, single-arm, monotherapy (10 mg/day PO)	MCL (n = 19) DLBCL (n = 47) Follicular grade 3 (n = 8) Other lymphomas (n = 3)	MCL 32% DLBCL 30% Follicular grade III 38% Other lymphomas 0%

Everolimus [39]	Phase I/II single-arm, monotherapy (5 or 10 mg/day PO)	MCL (n = 4) Other hematologic malignancies (n = 23)	MCL 0% Other 4%

Temsirolimus [40]	Phase II, single-arm, monotherapy (250 mg IV weekly)	MCL (N = 34)	38%

Temsirolimus [41]	Phase II, single-arm, monotherapy (25 mg IV weekly)	MCL (N = 27)	41%

Temsirolimus [42]	Phase III, monotherapy (175 mg IV weekly for 3 weeks, then 25 mg [n = 54] or 75 mg IV weekly [n = 54]) vs investigator-chosen chemotherapy (n = 54)	MCL (N = 162)	Temsirolimus 25 mg 6% Temsirolimus 75 mg 22% Investigator-chosen 2%

Ridaforolimus [43]	Phase II, single-arm, monotherapy (12.5 mg/day IV on days 1-5 every 2 weeks)	MCL (n = 9) Other hematologic malignancies (n = 43)	MCL 33% Others 5%

**Waldenström macroglobulinemia**			

Everolimus [44]	Phase II, single-arm, monotherapy (10 mg/day)	WM (N = 50)	42% (PR)

**Hodgkin lymphoma**			

Everolimus [45]	Phase II, single-arm, monotherapy (10 mg/day)	HL (N = 19)	HL 47%

**GVHD**			

Sirolimus [46]	Retrospective chart review, sirolimus conditioning (12 mg loading dose days 1-3, then 4 mg daily) vs standard conditioning	GVHD prophylaxis after HSCT for lymphoma (N = 126)^a^	Overall survival: Sirolimus 66% Standard conditioning 38%

Abbreviations: CR - complete response, DLBCL - diffuse large B-cell lymphoma, GVHD - graft-versus-host disease, HL - Hodgkin's lymphoma, HSCT - hematopoietic stem cell transplant, IV - intravenously, MCL - mantle-cell lymphoma, ORR - overall response rate, PO - orally, PR - partial response, WM - Waldenström macroglobulinemia.

^a^Overall survival reported as 3-year survival for patients receiving reduced intensity conditioning [46]

### Relapsed/refractory mantle cell lymphoma

The mTOR inhibitors, everolimus, temsirolimus, and ridaforolimus, have been evaluated in phase I and II trials of patients with relapsed/refractory MCL (Table 3). The efficacy and safety of everolimus monotherapy (10 mg/day for 4-week cycles) was evaluated in a phase II trial of 77 patients with relapsed aggressive NHL, including 19 patients with MCL and 47 patients with DLBCL [38]. The overall response rates were 30% (3 uCR/20 PR) for all patients, 32% for MCL, and 30% for DLBCL [38]. The median duration of response in patients achieving a CR or PR was 5.7 months, and of these patients, 5 remained progression-free at 12 months [38]. Monotherapy with everolimus was first evaluated in a phase I/II trial of 26 heavily pre-treated patients with relapsed or refractory MCL (n = 4) or other hematologic malignancies (n = 23) [39]. Everolimus modulated mTOR signaling in 6 of 9 patient samples within 24 hours as demonstrated by simultaneous inhibition of the downstream effectors, p70S6K and 4E-BP1 [39]. None of the 4 patients with MCL in this cohort achieved a clinical response to everolimus [39].

Temsirolimus has been studied in 2 phase I/II trials and 1 large phase III trial of patients with MCL (Table 3). The response rate to a 250-mg/week course of temsirolimus monotherapy in patients with advanced MCL was 38% (N = 34; 1 CR/12 PR) [40], which was similar to the 41% response rate (N = 27; 1 CR/10 PR) achieved by a similar cohort after treatment with a 10-fold lower dose of temsirolimus (25 mg/week) [41]. However, the 25-mg dose was associated with lower rates of hematologic toxicity, specifically thrombocytopenia [41]. Based on these findings, a large phase III trial of temsirolimus monotherapy was conducted. Patients with heavily pre-treated relapsed/refractory MCL (N = 162) were randomized to open-label treatment with investigator-chosen, pre-approved chemotherapy regimens or 1 of 2 regimens of temsirolimus monotherapy (175 mg/week for 3 weeks followed by either 25-mg or 75-mg weekly) [42]. The overall response rate was 6% for the 25-mg dose and 22% for the 75-mg dose, the latter being significantly higher (*p = 0.0019*) compared with investigator-chosen treatment (2%) [42]. Median progression-free survival was 3.4 months (25 mg), 4.8 months (75 mg), and 1.9 months (investigator-chosen; *p = 0.0009 *vs 75 mg) [42].

The anti-tumor activity of ridaforolimus, another intravenously administered mTOR inhibitor, has been evaluated in a phase II study of 52 patients with hematologic malignancies (including 9 patients with MCL) (Table 3) [43]. Patients were treated with ridaforolimus monotherapy 12.5 mg daily for days 1 to 5 every 2 weeks [43]. Of the 9 patients with MCL, 3 achieved a partial response for an overall response rate of 33% [43].

### Waldenström macroglobulinemia

A phase II trial of everolimus monotherapy (10 mg/day) was conducted in 50 patients with relapsed or relapsed/refractory Waldenström macroglobulinemia (WM) (Table 3) [44]. After a median treatment duration of 2 months (range: 1 to 10 months), 21 patients (42%) achieved a partial response. No patient had a CR. The median duration of response had not been reached by the time of publication, but 16 of the 21 patients continued to respond after a median 6.6-month follow-up (range: 1 to > 18.2 months) [44].

### Hodgkin lymphoma

The anti-tumor activity of everolimus monotherapy (10 mg/day) also was examined in a phase II study of 19 heavily pre-treated patients with relapsed HL (Table 3) [45]. The overall response rate was 47% (1 CR/8 PR), with a median duration of response of 7.1 months [45]. A multicenter trial has begun enrollment in the United States to confirm the activity of everolimus monotherapy in patients with relapsed/refractory HL (NCT01022996).

### Graft-versus-host disease

Armand and colleagues conducted a retrospective chart review of patients who underwent allogenic hematopoietic stem-cell transplantation for lymphoma [46]. Patients chosen for inclusion received graft-versus-host disease (GVHD) prophylaxis with the mTOR inhibitor sirolimus (12-mg loading doses on days 1-3 followed by 4 mg daily) or standard GVHD prophylaxis (cyclosporine or tacrolimus alone or in combination with methotrexate). Of 126 patients who received reduced intensity conditioning with sirolimus (n = 103) or with standard regimens (n = 23), the 3-year overall survival rate was 66% (*p = 0.007 *vs no sirolimus) in the sirolimus arm and 38% in the no-sirolimus group with a corresponding 3-year progression-free survival of 44% (*p = 0.001 *vs no sirolimus) and 17%, respectively [46].

### Diffuse large B-cell lymphoma

As previously noted, everolimus monotherapy has been evaluated in a phase II trial in patients with relapsed/refractory aggressive NHL, including 47 patients with DLBCL who achieved an overall response rate of 30% [38]. Several ongoing investigator-initiated trials are evaluating combining everolimus with other agents in the treatment of NHL. In addition, the PIvotaL Lymphoma triAls of RAD001 (PILLAR-2; NCT00790036), an ongoing phase III maintenance trial of everolimus in poor-risk patients with DLBCL who achieved a CR with R-CHOP chemotherapy, has begun enrolling patients (NCT00790036).

### Toxicity

Thrombocytopenia, neutropenia, and anemia are the most commonly reported hematologic toxicities reported during monotherapy with the mTOR inhibitors everolimus, temsirolimus, and ridaforolimus [38-44]. Not surprisingly, thrombocytopenia reported during temsirolimus 250 mg/week (100%) was more common than during treatment with the lower dose of 25 mg/week (39%) [40,41]. Differences in the rates of thrombocytopenia were less marked for temsirolimus 75-mg weekly (59%) versus 25-mg weekly (52%) [42]. Fatigue, mucositis, hyperglycemia, diarrhea, anorexia/weight loss, and hyperlipidemia are commonly occurring non-hematologic toxicities seen during mTOR inhibitor treatment [38-44]. Thrombocytopenia was a commonly reported reason for treatment delay or dose reduction [38,40,41,45].

Pulmonary toxicity can be observed with mTOR inhibitor therapy. Pulmonary symptoms, such as increased cough, dyspnea, and pleural effusion, have been reported during treatment with both everolimus and temsirolimus [38,42,44,45]. It is difficult to compare rates of pulmonary toxicity for the different mTOR inhibitors given non-standard descriptions of adverse events and the lack of direct, head-to-head studies. Nevertheless, rates of grade 3/4 dyspnea and other pulmonary symptoms were similar for everolimus (21%) and temsirolimus (16%) in 2 monotherapy studies [42,45]. Pulmonary symptoms associated with mTOR inhibition usually can be managed by interrupting treatment and restarting at a lower dose [38,44,45].

### Thalidomide Derivatives

The thalidomide derivative, lenalidomide, has been evaluated in a phase II multicenter study in patients with relapsed/refractory aggressive NHL [47]. Open-label treatment consisted of lenalidomide 25 mg daily for the first 21 days of every 28-day cycle; patients continued treatment for 52 weeks unless toxicity or disease progression occurred [47]. Of the 49 evaluable patients, 26 had DLBCL, 15 had MCL, 5 had grade 3 follicular lymphoma, and 3 had transformed low-grade lymphoma [47]. Overall response rates were 35% (4 uCR/2 CR/11 PR) for all 49 patients, 19% for DLBCL (2 uCR/1 CR/2 PR), and 53% for MCL (1 uCR/1 CR/6 PR) [47]. For the entire population of 49 patients, the median duration of response was estimated to be 6.2 months, and the median progression-free survival was 4.0 months [47]. The most common grade 3/4 hematologic toxicities were neutropenia, thrombocytopenia, and leukopenia [47]. Neutropenia, thrombocytopenia, and fatigue were the toxicities most likely to necessitate a reduction in dose [47].

Trial investigators updated the clinical outcome of the 15 patients with MCL [48]. The overall response rate remained at 53% (3 CR/5 PR), with 1 patient converting from a partial response to a complete response [48]. The median duration of response for the patients with MCL in the updated report was 13.7 months with a median progression-free survival of 5.6 months [48]. Hematologic and dose-limiting toxicities were consistent with that described in the initial report [47,48]. Based on these promising findings, a phase III multinational, placebo-controlled, first-line maintenance study of lenalidomide in patients with MCL is planned (NCT01021423).

## Discussion

Effective therapies for patients with lymphoma are urgently needed. Targeted therapy based on signal transduction pathway alterations detected in lymphomas offers the hope of reaching this goal. Monotherapy with the proteasome inhibitor, bortezomib, has shown efficacy in MCL, and combination therapy with conventional chemotherapy regimens also appears promising. Bortezomib does not appear to have appreciable anti-tumor activity in patients with DLBCL or HL. Demonstration of durable complete and partial responses to monotherapy with the mTOR inhibitors (everolimus, temsirolimus, and ridaforolimus) in phase I/II monotherapy trials support further study of this class of compounds in phase III trials.

Treatment with bortezomib or the mTOR inhibitors is relatively well-tolerated, especially in these cohorts of heavily pretreated patients. The most common dose-limiting toxicities associated with bortezomib (1.3 or 1.5 mg/m^2 ^twice weekly) were peripheral neuropathy, fatigue, and neutropenia. Similarly, the adverse events associated with the mTOR inhibitors were generally manageable; thrombocytopenia, neutropenia, and anemia were the most commonly reported hematologic toxicities. Starting doses of 10 mg/day for everolimus (with reductions to 5 mg/day if needed) and temsirolimus (175 mg/week for 3 weeks then 75 mg/week) are supported by the clinical trial data. Hypercholesterolemia or hypertriglyceridemia have been reported with the mTOR inhibitors [40,44,45], and one group of investigators recommends treating this adverse event with statins in patients continuing on long-term temsirolimus treatment [41].

Pulmonary toxicity associated with the mTOR inhibitors is an issue that needs to be carefully monitored and better understood. Dyspnea, cough, and pulmonary infiltrates have been observed in patients treated with everolimus and temsirolimus [38,42,44,45]. However, these symptoms may also be associated with infection or the tumor itself, both of which should be ruled out before attributing causality to the mTOR inhibitor. In our study of everolimus in patients with HL, we did not consider asymptomatic pulmonary infiltrates to be dose limiting; rather we reduced the dose of everolimus only when patients became symptomatic (eg, dyspnea on exertion or cough) [45].

The demonstrated activity of bortezomib in MCL, and the mTOR inhibitors everolimus and temsirolimus in DLBCL and MCL, suggests that these agents may one day have a place in the treatment armamentarium for aggressive lymphomas. Results of monotherapy trials are encouraging, and the use of bortezomib, everolimus, and temsirolimus in combination with chemotherapy regimens currently is being studied with the goal of maximizing the response and overall survival in patients with aggressive lymphomas.

## Abbreviations

DLBCL: diffuse large B-cell lymphoma; GVHD: graft-versus-host disease; HDACIs: histone deacetylase inhibitors; HL: Hodgkin lymphoma; MCL: mantle cell lymphoma; mTOR: mammalian target of rapamycin; NF-κB: nuclear factor-κB; NHL: non-Hodgkin lymphoma; PDGF: platelet-derived growth factor; PI3K: phosphoinositide 3-kinase; WM: Waldenström macroglobulinemia;

## Competing interests

TW has received research support from Novartis and Celgene for clinical trials. PBJ has served on an advisory board for Novartis (no personal compensation). RY is an employee of and has equity interest in Novartis. FC has served on advisory boards for Novartis.

## Authors' contributions

TW, PBJ, RY, and FC contributed to the conception of this manuscript and were involved in drafting and/or revising the manuscript. All authors have read and approved the final manuscript and have given final approval of the version to be published.
